# Immune-mediated microbial interference governs *Borrelia* colonization of the tick gut

**DOI:** 10.1016/j.isci.2026.115628

**Published:** 2026-04-24

**Authors:** Adnan Hodžić, Martin Kunert, Mia Juračić, Gorana Veinović, Ratko Sukara, Snežana Tomanović, David Seki, David Berry

**Affiliations:** 1 Centre for Microbiology and Environmental Systems Science, Department of Microbiology and Ecosystem Science, Division of Microbial Ecology, University of Vienna, Vienna 1030, Austria; 2 Institute for Medical Research, National Institute of Republic of Serbia, University of Belgrade, 11129 Belgrade, Serbia; 3 Joint Microbiome Facility of the Medical University of Vienna and the University of Vienna, Vienna 1030, Austria

**Keywords:** Immunology, Microbiology, Parasitology

## Abstract

The tick gut represents a dynamic environment where various ecological and molecular factors, including interactions between the tick innate immune system and its resident microbiota, govern the success of pathogen colonization. Yet, the mechanisms by which these microbial communities shape pathogen infection dynamics remain unclear. Here, we demonstrate that oral inoculation with the gut bacterium *Pseudomonas putida* modulates immune responses in *Ixodes ricinus* and restricts infection by *Borrelia afzelii*. Transcriptional profiling of immune-related genes revealed that *Pseudomonas* specifically induces the expression of the gene encoding the antimicrobial peptide defensin, reinforcing epithelial defenses without extensive activation of canonical signaling pathways. Functional assays demonstrated that *Pseudomonas* impedes *Borrelia* colonization through a host-mediated mechanism rather than direct microbial antagonism. These findings reveal a microbiota-driven immune pathway that constrains pathogen persistence in the tick gut and provide insights into the tripartite interplay between the tick host, its microbiota, and borrelial pathogens.

## Highlights

• Expression patterns of immune genes vary in response to different bacterial taxa
• Oral inoculation with *Pseudomonas* reduces *Borrelia afzelii* load in the tick gut
• *Pseudomonas* upregulates tick defensin, which inhibits the growth of *Borrelia*
• *Pseudomonas* increases resistance to *Borrelia* infection in wild-caught ticks

## Graphical abstract

**Figure:**
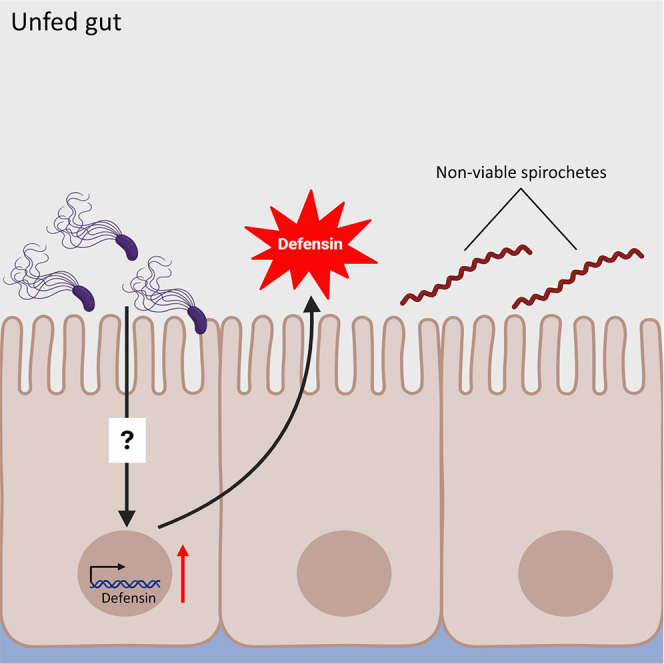

## Introduction

Ixodid ticks are obligate hematophagous ectoparasites and important vectors for a wide range of bacterial, viral, and protozoan pathogens that affect both human and animal health worldwide.^1^ In Europe, *Ixodes ricinus* serves as the principal vector of *Borrelia burgdorferi* sensu lato, the etiological agent of Lyme borreliosis (LB), which remains the most prevalent vector-borne disease in temperate regions of the Northern Hemisphere.^2^ After being acquired during feeding on an infected vertebrate host, *Borrelia* must navigate the hostile microenvironment of the tick gut, where it can persist for extended periods before eventually migrating to the salivary glands during a subsequent blood meal. The tick gut therefore serves as a selective bottleneck in which ecological and molecular factors, including interactions between the tick innate immune system and its commensal microbiota, govern the efficiency of pathogen colonization and subsequent transmission.^3^

The microbiota of disease-transmitting arthropods is increasingly acknowledged as a major factor in determining vector competence. Mounting evidence indicates that it can influence pathogen infection by engaging in competitive interactions and/or by modulating the host innate immune system and epithelial barrier integrity.^4^^,^^5^^,^^6^^,^^7^^,^^8^ Ticks harbor relatively complex and abundant microbial communities, and several experimental and field studies suggest that their microbiota may either facilitate or hinder pathogen acquisition and persistence.^9^^,^^10^^,^^11^^,^^12^^,^^13^^,^^14^^,^^15^^,^^16^^,^^17^^,^^18^ Besides intracellular symbionts, the resident gut microbiota of *Ixodes* ticks harbors bacteria such as *Pseudomonas*, *Bacillus*, and *Staphylococcus*, which are acquired from the environment and vertebrate hosts.^9^^,^^11^^,^^17^^,^^18^^,^^19^^,^^20^^,^^21^ Perturbation of microbial communities, either through antibiotic exposure, environmental changes, or anti-microbiota vaccination, has been found to influence *Borrelia* infection dynamics in engorged ticks.^12^^,^^22^^,^^23^^,^^24^^,^^25^^,^^26^

Ticks exhibit a refined capacity to balance immune tolerance toward commensal or beneficial microbes with protective immune responses against harmful pathogens.^27^ This finely tuned equilibrium is essential for maintaining gut homeostasis, as excessive immune activation may result in tissue damage or dysbiosis, whereas insufficient immune defense can allow pathogen proliferation.^8^ Lacking adaptive immunity, ticks exclusively depend on innate defenses consisting of tightly regulated humoral and cellular responses. Cellular defenses include phagocytosis, encapsulation, and nodulation of invading pathogens, whereas the humoral response relies on various pattern recognition proteins and effector molecules.^28^ Pathogen recognition and subsequent signaling occur through conserved pathways such as Janus kinase/signal transducer and activator of transcription (JAK/STAT), Toll, and immune deficiency (IMD), which coordinate the antimicrobial response through the production of antimicrobial peptides (AMPs), reactive oxygen species (ROS), reactive nitrogen species (RNS), and other immune effectors.^28^ Notably, these immune mechanisms are not only activated in response to invading pathogens but also function continuously to support microbial balance in the gut.^27^^,^^29^ While substantial progress has been made in elucidating tick immune responses to transmitting pathogens,^30^ the molecular mechanisms by which the gut microbiota interact with tick immunity and possibly affect *Borrelia* colonization remain poorly understood.

In this study, we investigated the influence of gut bacteria on the expression of immune-related genes in *I. ricinus*. Furthermore, we identified tick defensin as a key immune effector and characterized its functional role in maintaining gut microbial homeostasis and modulating tick susceptibility to *Borrelia* infection. Our findings demonstrate that an immune response elicited by *Pseudomonas* impedes *Borrelia afzelii* colonization. Collectively, this study provides insights into the intricate interplay between the tick immune system, resident gut microbiota, and borrelial pathogens. By emphasizing the significance of these interactions as potential intervention points, this work could facilitate microbiome-based strategies to disrupt the transmission cycle of LB and promote the development of targeted control measures.

## Results

### Gut immune signaling pathways remain largely unresponsive to bacterial challenges

**Figure 1:**
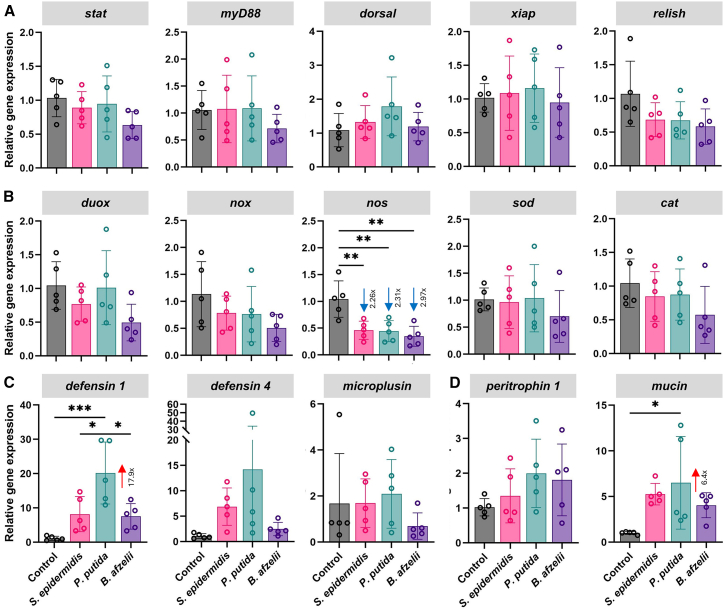
Transcriptional profiles of genes involved in tick immunity (A–D) Relative expression of representative genes associated with (A) major components of immune signaling pathways, (B) redox homeostasis, (C) AMP production, and (D) physical protection in guts of control ticks (PBS) and ticks fed *S. epidermidis*, *P. putida*, or *B. afzelii*. Gene expression was quantified by RT-qPCR at 8 h post-infection and normalized to tick *elf-1a*. Each dot represents a pool of three guts. Data are presented as the mean ± SD of two technical replicates. Statistical significance was evaluated using one-way ANOVA or the Kruskal-Wallis test (*cat*, *defensin 4*, *microplusin*), depending on the data distribution. ^∗^*p* < 0.05; ^∗∗^*p* < 0.01; ^∗∗∗^*p* < 0.001. Gene names: *duox*, dual oxidase; *nox*, NADPH oxidase; *nos*, nitric oxide synthase; *sod*, superoxide dismutase; cat: catalase.

To characterize gut-specific immune responses triggered by different bacterial taxa and to identify factors contributing to gut colonization resistance, adult female ticks from a laboratory colony were challenged via capillary feeding with either the skin-associated Gram-positive bacterium *Staphylococcus epidermidis*, the environmental Gram-negative bacterium *Pseudomonas putida*, or the atypical Gram-negative pathogen *B. afzelii*. The transcriptional profiles of selected genes representing major components of the JAK/STAT (*stat*), Toll (*myD88*, *dorsal*), and IMD (*xiap*, *relish*) immune signaling pathways, as well as genes associated with redox homeostasis (*duox*, *nox*, *nos*, *sod*, *cat*), antimicrobial defenses (*defensin 1*, *defensin 3*, *microplusin*), and physical gut protection (*peritrophin 1*, *mucin*), were analyzed by quantitative reverse transcription PCR (RT-qPCR) at 8 h post-feeding. Interestingly, expression levels of immune pathway genes did not differ significantly in ticks exposed to any of the tested bacterial species compared to phosphate-buffered saline (PBS) controls (Figure 1A). Among the genes involved in redox homeostasis, *nos*, which is responsible for producing the antimicrobial molecule nitric oxide, was found to be consistently and significantly downregulated in all experimental groups (Figure 1B). Its suppression following bacterial infections, alongside the unaltered expression levels of other redox genes (*duox*, *nox*, *sod*, *cat*), suggests reduced RNS production. To further assess the levels of ROS and RNS in the guts of infected ticks, fluorescence microscopy was performed using the probe dihydroethidium (DHE) for superoxide detection and 5,6-diaminofluorescein diacetate (DAF-2DA) for nitric oxide detection. These results were consistent with the gene expression data and support the observation that bacterial exposure did not induce pronounced oxidative or nitrosative stress responses in the infected guts (Figures S1A and S1B), suggesting that these pathways are unlikely to play a major role in gut resistance to early bacterial infections. However, a subtle increase in ROS production was seen in salivary glands upon feeding, indicating a tissue-specific oxidative response (Figure S1A). Notably, *B. afzelii* infection resulted in a general but not significant reduction in transcript levels across nearly all tested genes compared with the other bacterial treatments (Figures 1A–1D).

Analysis of AMPs revealed more specific transcriptional responses. Specifically, *defensin 1* was upregulated in response to all tested bacteria, although the increase was statistically significant only in *P. putida*-infected ticks. The transcript levels of *defensin 4* and *microplusin* were not affected (Figure 1C). Similarly, *mucin* was significantly upregulated following *P. putida* infection (Figure 1D). Collectively, these findings indicate that *P. putida* triggers the most pronounced epithelial response among the tested bacterial species, characterized by simultaneous activation of antimicrobial effectors and reinforcement of physical gut protection. The overall transcriptional landscape implies a restrained yet coordinated immune modulation shaped by bacterial taxonomic identity.

### Commensal gut bacteria modulate the colonization ability of *Borrelia afzelii*

**Figure 2:**
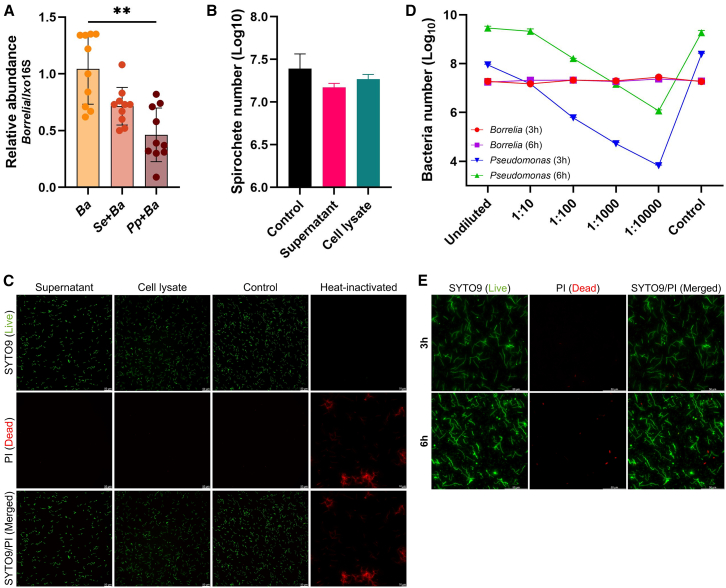
*Pseudomonas putida* interferes with *B. afzelii* colonization of the tick gut (A) Relative abundance of *B. afzelii* in the guts of female ticks co-infected with *S. epidermidis* (*Se*+*Ba*) or *P. putida* (*Pp*+*Ba*) at 7 h post-*Borrelia* infection was evaluated by qPCR and compared with ticks infected with *B. afzelii* alone (*Ba*). Each dot represents an individual tick gut. Data were normalized to the tick mitochondrial 16S rRNA gene and are presented as the mean ± SD of two technical replicates. Statistical significance was determined using the Kruskal-Wallis test (^∗∗^*p* < 0.01). (B) The number of *B. afzelii* spirochetes in cell cultures was measured by qPCR after 24 h of incubation with cell-free supernatant or cell lysate from an overnight culture of *P. putida*. Untreated *Borrelia* culture served as a positive control. Data represent the mean ± SD of two technical replicates. Statistical significance was determined using one-way ANOVA (*p* ˃ 0.05). (C) Viability of *B. afzelii* spirochetes was assessed using SYTO9/PI staining to distinguish live (green) and dead (red) cells after 24 h of incubation with *P. putida* supernatant or cell lysate. Heat-inactivated spirochetes (70°C for 10 min) served as a positive control. Scale bars, 10 μm (heat-inactivated), 50 μm. (D) qPCR quantification of *B. afzelii* and *P. putida* after 3 and 6 h of co-cultivation using different *P. putida* inoculum dilutions. Pure cultures of each species were used as positive controls. Data represent the mean ± SD of two technical replicates. (E) Viability of *B. afzelii* and *P. putida* after 3 and 6 h of co-cultivation, assessed by SYTO9/PI staining and microscopy. Scale bars, 50 μm.

The tick gut is the primary site of infection for pathogens acquired during blood feeding and serves as the environment where these pathogenic organisms interact with the resident gut microbiota.^7^ To investigate whether gut bacteria influence *Borrelia* colonization of the gut, ticks were fed either PBS alone (control) or suspensions containing *S. epidermidis* or *P. putida*, followed by infection with the wild-type *B. afzelii* strain. Each tick received a standardized inoculum containing equal numbers of bacterial cells to ensure experimental consistency and minimize potential bias in bacterial uptake and feeding efficiency. All ticks survived the procedure, and qualitative assessment of post-feeding activity (e.g., mobility and questing activity) did not indicate any deviation from normal tick behavior. Borrelial colonization dynamics were assessed by qPCR analysis of individual guts 7 h post-*Borrelia* infection. The results showed that prior exposure of ticks to gut bacteria led to a reduced *Borrelia* burden compared with ticks infected with *Borrelia* alone (Figure 2A). This reduction was particularly pronounced and statistically significant in ticks co-infected with the environmental bacterium *P. putida*, whereas those exposed to skin-associated *S. epidermidis* showed only a modest, non-significant decrease in spirochetal load (Figure 2A). To assess the persistence of the introduced bacteria, genus-specific qPCR assays were performed, revealing complete clearance of staphylococci (data not shown) and persistent gut colonization of *Pseudomonas* (Figure S2A).

To investigate whether this inhibitory effect resulted from direct microbial antagonism, an *in vitro* assay was performed in which *B. afzelii* was incubated with cell-free supernatants or cell lysates prepared from an overnight culture of *P. putida*. After 24 h of incubation, no detectable alterations in *B. afzelii* growth or viability were found by qPCR and microscopy relative to the untreated control (Figures 2B and 2C). Moreover, cocultivation of live *B. afzelii* with *P. putida* for 3 or 6 h did not affect the growth kinetics or viability of either species, indicating the absence of direct bactericidal or inhibitory interactions under the tested conditions (Figures 2D and 2E). Based on these findings, we speculate that the inhibitory effect of *P. putida* on *Borrelia* colonization is likely not driven by direct interbacterial antagonism but rather by indirect, host-mediated mechanisms.

### Defensin 1 exhibits selective antimicrobial activity and contributes to *B. afzelii* control in ticks

**Figure 3:**
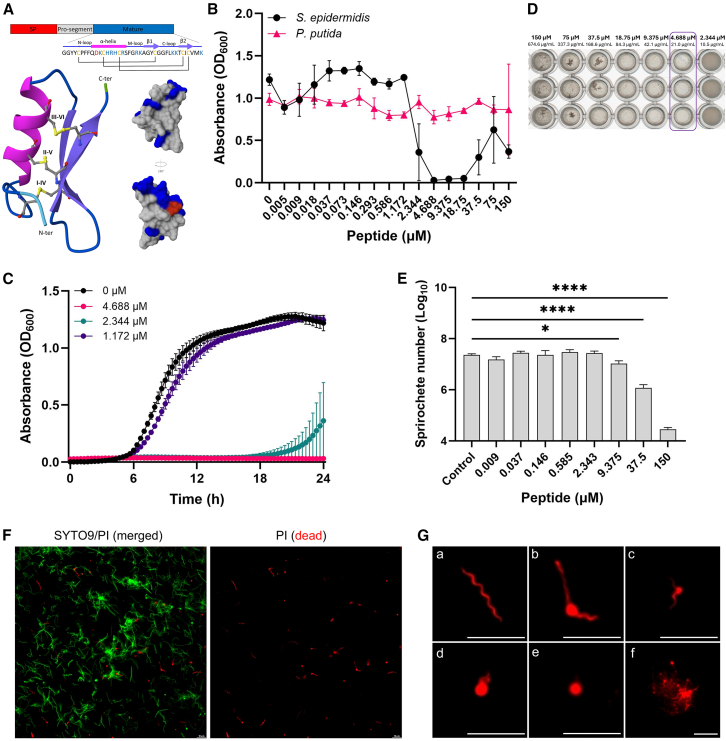
Defensin 1 exhibits selective antimicrobial activity (A) Domain architecture and the predicted 3D structure of mDef1 (alphafoldserver.com). The α-helix (magenta) and β-sheets (violet) are stabilized by three disulfide bonds formed between six conserved cysteine residues (Cys1–Cys4, Cys2–Cys5, Cys3–Cys6). The surface representation highlights negatively (red) and positively (blue) charged residues, demonstrating the overall cationic nature of the peptide surface. (B–D) Growth inhibition assays. The OD_600_ of *S. epidermidis* and *P. putida* was measured after 24 h of incubation with 2-fold serial dilutions of mDef1. Data are presented as mean ± SD of three technical replicates. (C) Temporal growth dynamics of *S. epidermidis*. (D) Visible peptide precipitates were observed at the three highest mDef1 concentrations, which likely contributed to the elevated OD_600_ values detected in the growth curves after 24 h of incubation (see Figure 3B). Wells containing the minimum inhibitory concentration (MIC) of mDef1 are outlined. (E) Concentration-dependent borreliacidal activity of mDef1. Spirochete loads were quantified by qPCR after 24 h of incubation with 4-fold serial dilutions of mDef1. Data are presented as the mean ± SD of two technical replicates. Statistical significance was determined using one-way ANOVA (^∗^*p* < 0.05; ^∗∗∗∗^*p* < 0.0001). (F) Exposure to mDef1 alters *B. afzelii* morphology and reduces viability. Spirochetes exposed to mDef1 exhibit marked morphological changes and decreased viability, as revealed by SYTO9/PI staining and fluorescence microscopy. The spirochetes aggregated into clusters and shifted into persister forms (round bodies). Scale bars, 10 μm. (G) DEAD/LIVE bacterial viability assay of *B. afzelii* spirochetes following 24 h incubation with mDef1. PI-positive pleomorphic forms indicate compromised outer membranes in flat-wave spirochetes (a), spirochetes with blebs (b, c), round bodies (d, e), and aggregates (f). Scale bars, 10 μm.

Since a direct microbial interaction between *B. afzelii* and *P. putida* could not be demonstrated in our previous experiments, we hypothesized that *P. putida* may indirectly influence *Borrelia* colonization by inducing *defensin 1* expression. Consistent with this hypothesis, *defensin 1* transcript levels were significantly elevated in ticks co-infected with *Pseudomonas* compared with other groups (Figure S2B). To clarify the role of defensin 1 in gut colonization resistance, we evaluated the antimicrobial activity of synthetic mature defensin 1 (mDef1) against *S. epidermidis*, *P. putida*, and *B. afzelii*. mDef1 is a small, positively charged peptide that features one α-helix and two β-sheets, stabilized by three disulfide bonds connecting six conserved cysteine residues (Figure 3A). *In vitro* inhibition assays demonstrated that mDef1 completely suppressed *S. epidermidis* growth at a concentration of 4.688 μM (21 μg/mL), whereas *P. putida* showed no susceptibility at any tested peptide concentration (Figures 3B–3D). The observed increase in optical density (OD_600_) at the three highest peptide concentrations after 24 h of incubation likely resulted from peptide precipitation (Figures 3B and 3D).

We next evaluated the inhibitory potential of mDef1 on the growth of *B. afzelii*. For this purpose, spirochetes were cultured in media supplemented with different peptide concentrations and compared with untreated controls. After 24 h of incubation, qPCR analysis revealed a concentration-dependent reduction in *B. afzelii* numbers, with a statistically significant decrease observed at concentrations of 9.375 μM (42 μg/mL) or higher (Figure 3E). mDef1 also induced notable morphological changes and impaired *B. afzelii* viability even at lower concentrations (≤0.293 μM) (Figures 3F and 3G). The cells frequently aggregated and shifted from their typical flat-wave morphology to spirochetes with outer membrane blebs and round body forms (Figures 3F and 3G). Bleb formation represents an early morphological manifestation of membrane alteration and serves as a hallmark of bacterial adaptive responses to various stress conditions, including oxidative stress, antimicrobial exposure, and host complement activation.^31^^,^^32^^,^^33^^,^^34^ In this context, fluorescence microscopy revealed that mDef1 compromised membrane integrity of multiple *B. afzelii* morphotypes, as evidenced by the accumulation of propidium iodide (PI) within the cells^34^^,^^35^ (Figures 3F and 3G). To further assess the specificity of mDef1, peptide folding was disrupted by treatment with the reducing agent dithiothreitol (DTT). Reduction of disulfide bonds by DTT significantly decreased the antimicrobial activity of mDef1 against *B. afzelii* (Figure S3), thereby supporting the specificity of its activity.

**Figure 4:**
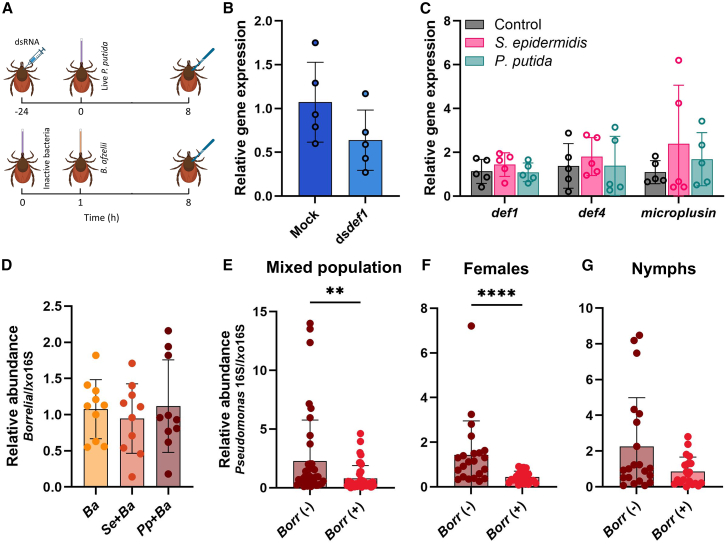
Inverse correlation between *Pseudomonas* abundance and *Borrelia* infection (A) Schematic overview of the gene silencing (top) and the tick feeding experiments with heat-inactivated bacteria (bottom). Figure created with BioRender (biorender.com). (B) Efficacy of *defensin 1* gene silencing in female ticks. *Defensin 1* transcript levels were quantified by RT-qPCR at 8 h post-infection with *P. putida* in mock- and ds*def1*-injected ticks. Expression levels were normalized to the tick housekeeping gene *elf-1a*. Data are presented as mean ± SD from two technical replicates, with each data point representing a pool of three guts. Statistical comparison between groups was performed using a two-tailed *t* test (*p* ˃ 0.05). (C) Relative gene expression of AMP genes in ticks fed heat-inactivated bacterial cells, normalized to the tick *elf-1a*. Each data point represents a pool of three guts. One-way ANOVA was used to determine statistical significance (*p* > 0.05). (D) Quantification of *B. afzelii* burden in guts of female ticks exposed to heat-inactivated bacterial cells. *Borrelia* loads were measured by qPCR at 7 h post-*Borrelia* infection. Data were normalized to the tick mitochondrial 16S rRNA gene and compared to the single-infection group. Each dot represents an individual tick gut. Results are shown as mean ± SD from two technical replicates. Statistical significance was assessed using one-way ANOVA (*p* > 0.05). (E–G) Relative abundance of *Pseudomonas* species in field-collected *I. ricinus*. Genus-specific qPCR was employed to quantify *Pseudomonas* levels in individual tick guts. Data were normalized to the tick mitochondrial 16S rRNA gene and compared between uninfected and *Borrelia*-infected nymphs and females. Results are presented as mean ± SD. Statistical comparisons between groups were conducted using the Mann-Whitney *U* test (^∗∗^*p* < 0.01; ^∗∗∗∗^*p* < 0.0001).

Initial attempts to investigate the functional role of defensin 1 in *B. afzelii* colonization *in vivo* through RNA interference (RNAi)-mediated gene knockdown were unsuccessful. Quantitative analysis performed 8 h after oral administration of *P. putida* showed no significant reduction in transcript levels in ticks injected with double-stranded RNA targeting *defensin 1* (ds*def1*) compared with control ticks (Figures 4A and 4B), indicating inefficient silencing. To improve knockdown efficiency, we extended the post-feeding period to 24 h. This, however, resulted in markedly reduced tick survival, precluding further bacterial co-feeding assays. Given these constraints, we adopted an alternative approach that involved oral administration of heat-inactivated (autoclaved) bacterial cells to ticks. This treatment did not trigger the upregulation of AMPs, including *defensin 1* (Figure 4C), and the inhibitory effect of *P. putida* on *B. afzelii* colonization was abolished (Figure 4D). Collectively, these findings highlight defensin 1 as a key effector molecule in antimicrobial gut defense, contributing to immune-mediated regulation of the tick microbial community and potentially enhancing its resistance to *B. afzelii* infection.

### *Pseudomonas* abundance correlates with the resistance of field-collected ticks to *Borrelia* infection

To examine whether *Pseudomonas* species impair gut infection with *Borrelia* under natural conditions, we analyzed microbial loads in questing *I. ricinus* nymphs and adult females collected from two *Borrelia*-endemic sites in Vienna.^36^ In total, 140 ticks were screened for *Borrelia burgdorferi* sensu lato using PCR, of which 43 (30.7%) tested positive. To evaluate the relationship between *Pseudomonas* abundance and *Borrelia* infection status, we performed genus-specific qPCR and compared *Pseudomonas* DNA levels between *Borrelia*-infected ticks and an equal number of randomly selected uninfected ticks. Across the entire tick population, uninfected ticks exhibited significantly higher *Pseudomonas* levels compared with infected ones (Figure 4E). This inverse association was most pronounced in adult females (Figure 4F). Although a similar trend was observed in nymphs, the difference between infected and uninfected groups was not statistically significant (Figure 4G). These results mirror the outcomes of our laboratory infection experiments and suggest that *Pseudomonas* species may naturally constrain *Borrelia* colonization in wild tick populations, thereby enhancing tick resistance to *Borrelia* infection under field conditions.

## Discussion

This study sheds light on gut-specific immune responses to different bacterial taxa and uncovers a previously uncharacterized mechanism of immune-mediated microbial interference in ticks. Specifically, we demonstrate that exposure to a gut commensal triggers an innate immune response that interferes with *B. afzelii* colonization. By correlating *Pseudomonas* abundance with reduced *Borrelia* infection in both experimentally infected and field-collected ticks, we established a functional link between the resident gut microbiota, epithelial immune activation, and pathogen persistence, suggesting a direct role of gut commensals in shaping tick vector competence.

Transcriptomic analysis showed a lack of robust activation of the JAK/STAT, Toll, and IMD signaling pathways in response to bacterial challenges. This subdued immune response aligns with previous transcriptomic studies^29^^,^^37^ and may reflect an evolutionary adaptation that enables ticks to accommodate beneficial bacteria while preventing excessive immune activation, which could compromise gut epithelial integrity. Nevertheless, *P. putida* triggered a subtle yet coordinated epithelial response, characterized by increased expression of *defensin 1* and *mucin* genes. The induction of *defensin 1* indicates a localized antimicrobial defense that may function independently of classical immune pathways or through alternative regulatory mechanisms. Supporting this notion, recent research has indicated that the tick gut relies on a set of continuously expressed immune effectors, including defensins, to provide baseline protection against environmental microbes during extended fasting periods.^29^ Simultaneous upregulation of *mucin*, which encodes a structural glycoprotein that contributes to both the physical and biochemical functions of the gut barrier,^38^ further suggests enhancement of epithelial defense mechanisms. The consistent downregulation of *nos* in all infection groups and stable expression of other redox-associated genes point to tightly regulated oxidative and nitrosative processes during early bacterial infection. Such precise modulation of redox processes may reflect a conserved mechanism that bacteria exploit for immune evasion and persistence,^39^^,^^40^ particularly considering the potent borreliacidal effects of ROS and RNS.^31^^,^^41^^,^^42^^,^^43^ In contrast to the systemic immune activation typically observed in insects,^8^^,^^44^^,^^45^^,^^46^^,^^47^ ticks seem to mount a more restrained and targeted immune response, reflecting their unique strategy to balance microbial regulation with gut homeostasis.

Our co-infection experiments demonstrated that *P. putida* substantially inhibited *B. afzelii* colonization, whereas *S. epidermidis* caused only a mild reduction in *B. afzelii*. These findings highlight that the modulatory effects of gut bacteria on *Borrelia* establishment and survival are taxon-specific. A similar observation has been reported in nymphal *I. ricinus* ticks co-infected with *B. afzelii* CB43 and *Escherichia coli* BL21 strains, introduced either orally or via microinjection.^23^ In that study, the authors proposed that inhibition of *Borrelia* colonization resulted from a microbiota shift toward a distinct community state divergent from that induced by the pathogen itself. While this explanation is plausible, we hypothesized that additional mechanisms, such as direct microbial antagonism or host-mediated immune modulation, may contribute to bacterial interference. All *Borrelia* species lack interbacterial effector and immunity genes commonly used by other bacteria to compete with cohabiting microbes.^9^ Our results, however, indicated that neither extracellular metabolites nor cellular components of *P. putida* influenced *Borrelia* viability, implying a limited capability of this bacterium to impose direct inhibitory pressure on the spirochetes. The *P. putida* KT2440 strain used in our study possesses a functional type VI secretion system (T6SS) that injects toxic effector proteins into neighboring competitor microbes through a contact-dependent mechanism.^48^^,^^49^^,^^50^ Nonetheless, cocultivation with *B. afzelii* did not show any direct inhibitory or bactericidal effects, suggesting that *Borrelia* may employ alternative mechanisms to persist within polymicrobial environments. Taken together, our results suggest that the suppression of *Borrelia* colonization by *P. putida* is primarily indirect and likely depends on interactions with the tick immune system, rather than on direct competition or secretion of antimicrobial factors.

This hypothesis was further supported by functional *in vitro* experiments demonstrating that mDef1 exhibits potent, concentration-dependent antimicrobial activity against *B. afzelii* but not against *P. putida*. While this study reports the borreliacidal activity of defensin 1, the anti-*Borrelia* properties of other tick defensins have been previously documented.^25^^,^^51^^,^^52^ The selective resistance of *P. putida* likely explains its persistence in the tick gut despite triggering *defensin 1* expression. This induced defensin, in turn, targets *Borrelia* spirochetes, which appear to be more susceptible to membrane disruption by the peptide. *Borrelia* is an atypical Gram-negative bacterium that lacks both lipopolysaccharides and peptidoglycans in its cell envelope and instead possesses immunoreactive glycolipids.^32^^,^^53^ The distinct outer membrane architectures of *Borrelia* and the Gram-negative bacterium *Pseudomonas* likely account for their differing susceptibilities to defensins, given that defensin activity and electrostatic interactions are known to depend on the composition and surface charge of target cell membranes.^54^ These interactions illustrate a form of indirect microbial competition in ticks that is driven by host immunity and is consistent with the concept of immune-mediated microbial interference observed in other arthropod vectors. A well-characterized example of this phenomenon occurs in mosquitoes, where the Gram-negative bacterium *Wolbachia* boosts the basal immune response, enhancing their resistance to pathogens such as dengue virus, Zika virus, and malaria parasites.^55^^,^^56^^,^^57^^,^^58^

The observed negative correlation between *Pseudomonas* abundance and *Borrelia* infection in field-collected *I. ricinus* supports previous reports^9^^,^^17^ and further underscores the ecological relevance of our laboratory findings. This inverse relationship suggests that naturally occurring variations in tick microbiota may partly account for the heterogeneous infection rates observed among tick populations and across geographic regions.^16^^,^^36^^,^^59^^,^^60^ Adult females, which showed the strongest negative association, are likely subject to greater environmental exposure to soil-associated bacteria such as *Pseudomonas*, potentially enhancing their immune preparedness. This notion is further reinforced by the finding that *defensin 1* is expressed at significantly higher levels in questing *I. ricinus* females than in nymphs.^61^ Such environmentally acquired microbes may therefore act as natural modulators of vector competence that fluctuate seasonally or geographically according to local microbial community composition.

In conclusion, this study uncovers a critical role of gut bacteria in modulating tick innate immunity and controlling pathogen colonization. The discovery that *Pseudomonas* species can elicit defensin-mediated suppression of *Borrelia* establishes a direct mechanistic link between microbial community composition and vector competence in *I. ricinus*. These findings broaden the conceptual framework of immune-mediated microbial interference in ticks and highlight the potential of exploiting resident bacterial taxa to mitigate pathogen transmission. However, involvement of additional immune effectors beyond those examined here cannot be excluded, and the contribution of other microbial taxa to immune priming or antagonism warrants further investigation. Future research should thus aim to elucidate broader molecular crosstalk between resident microbiota and tick immune pathways, define bacterial determinants driving immune activation, and determine how environmental variables influence tripartite interactions under natural conditions.

### Limitations of the study

Although our study offers valuable conceptual insights into tick-microbiota-pathogen interactions, several limitations should be acknowledged. First, all experiments were performed under laboratory conditions, which may not fully reflect the complexity and variability of the natural environment. Such controlled settings could have favored the growth of certain bacterial taxa, potentially biasing the observed microbial dynamics. To mitigate this, we also analyzed ticks collected from the field, and the microbial interactions in these samples largely reflected those observed in the laboratory. Second, gene expression analysis was conducted at a single time point, which may have missed important regulatory processes and dynamic host responses that occur at later stages of infection. Finally, our experimental framework captured only short-term effects of co-infection on *Borrelia* abundance, but the field-derived data revealed patterns indicative of a sustained reduction in *Borrelia* burden in the presence of *Pseudomonas* species. Future studies should examine multiple post-infection time points to achieve a more comprehensive and ecologically relevant understanding of these interactions.

## Resource availability

### Lead contact

Further information and requests should be directed to and will be fulfilled by the lead contact, Adnan Hodžić (adnan.hodzic@univie.ac.at).

### Materials availability

All material generated during this study will be made available upon request.

### Data and code availability

• All data generated in this study will be shared by the lead contact upon request.
• This paper does not report original code.
• Any additional information required to reanalyze the data reported in this paper is available from the lead contact upon request.

## Acknowledgments

This research was funded in whole or in part by the 10.13039/501100002428Austrian Science Fund (grant DOI: 10.55776/P36130). For open access purposes, the author has applied a CC BY public copyright license to any author accepted manuscript version arising from this submission. The authors would also like to thank Georgi Nikolov and Viktoria Cizek for their excellent technical assistance. The work of G.V., R.S., and S.T. was supported by the Ministry of Science, Technological Development and Innovation of the Republic of Serbia (contract number 451-03-136/2025-03/200015 with Institute for Medical Research, University of Belgrade, National Institute of the Republic of Serbia).

## Author contributions

A.H.: conceptualization, data curation, formal analysis, funding acquisition, investigation, methodology, resources, validation, visualization, writing – original draft. M.K.: data curation, formal analysis, investigation, methodology, validation, writing – review and editing. M.J.: data curation, formal analysis, investigation, methodology, writing – review and editing. G.V.: data curation, formal analysis, investigation, methodology, writing – review and editing. R.S.: data curation, formal analysis, investigation, methodology, writing – review and editing. S.T.: investigation, methodology, resources, writing – review and editing. D.S.: formal analysis, methodology, writing – review and editing. D.B.: supervision.

## Declaration of interests

The authors declare no competing interests.

## Declaration of generative AI and AI-assisted technologies in the writing process

During the preparation of this work, the authors used the Perplexity AI tool to improve the readability, grammar, and language of the manuscript. After using this tool, the authors reviewed and edited the content as needed and take full responsibility for the content of the published article.

## STAR★Methods

### Key resources table

**Table:** 

REAGENT or RESOURCE	SOURCE	IDENTIFIER
**Bacterial strains**		

*Staphylococcus epidermidis*	Hodžić et al.^18^	LTG-5
*Pseudomonas putida*	DoME collection	KT2440
*Borrelia afzelii*	Ćakić et al.^62^	RS 163_11i

**Biological samples**		

*Ixodes ricinus* nymphs and females	Wild-caught, Vienna, Austria	N/A; collected in 2023

**Chemicals, peptides, and recombinant proteins**		

Ethanol	Thermo Fisher Scientific	Cat# 16606002
USB Dithiothreitol (DTT), 0.1M Solution	Thermo Fischer Scientific	Cat# 707265ML
Synthetic defensin 1 (mDef1)	GenScript	U4651WHBG0

**Critical commercial assays**		

Total RNA Purification kit	Norgen Biotek	Cat# 17200-NB
2-mercaptoethanol	Sigma-Aldrich	Cat# M6250
Qubit RNA Broad Range Assay Kit	Thermo Fisher Scientific	Cat# Q10210
TURBO DNA-free Kit	Invitrogen	Cat# AM1907
High-Capacity cDNA Reverse Transcription Kit	Applied Biosystems	Cat# 4368814
iQ SYBR Green Supermix	Bio-Rad	Cat# 1708882
QIAamp DNA Mini Kit	Qiagen	Cat# 51304
GoTaq Probe qPCR Mastermix	Promega	Cat# A6101
Monarch Spin PCR & DNA Cleanup Kit	New England Biolabs	Cat# T1130S
MEGAscript RNAi Kit	Ambion	Cat# AM1626
DHE (Dihydroethidium)	MedChemExpress	Cat# HY-D0079
DAF-2DA (4,5-Diaminofluorescein diacetate)	MedChemExpress	Cat# HY-D0032
LIVE/DEAD™ BacLight^TM^ Bacterial Viability Kit	Invitrogen	Cat# L7012

**Experimental models: Organisms/strains**		

*Ixodes ricinus* nymphs and females	Insect Services GmbH	N/A

**Oligonucleotides**		

See Table S1	supplemental information	N/A

**Software and algorithms**		

ImageJ	National Institute of Health	v1.80.345/1.54g
AlphaFold Server	alphafoldserver.com	N/A
CFX Manager	Bio-Rad	v2.1
Leica Application Suite LAS X	Leica Microsystems	v3.7.6.25997
GraphPad Prism	GraphPad	v10

**Other**		

BSK-H Complete Medium	Sigma-Aldrich	Cat# B8291
LB Broth (Lennox)	Thermo Fisher Scientific	Cat# H26760.36
Mueller-Hinton Agar 2	Merck Millipore	Cat# 97580
Microcaps, Volume 5,0 μl, Length 32 mm	Drummond	Cat# 0810005
Olympus SZ61 Stereomicroscope	Olympus SZ61	Cat# SZ61-RT
Incubator	Pol-Eko-Aparatura	Cat# UM 400
Surgical blades No. 11	Fischer Scientific	Cat# 12697896
Fine-tipped forceps (12 cm)	Medizinische Instrumente May	Cat# PI-0039
FastPrep®-24 5G bead beating grinder	MP Biomedicals	N/A
innuSPEED Lysis Tubes P	IST Innuscreen GmbH	Cat# 845-CS-1020050
Qubit 4 Fluorometer	Invitrogen	Cat# Q33226
CFX96 Real-Time PCR cycler	Bio-Rad	N/A
Microliter syringe 33-gauge	Hamilton	N/A
Leica DMi8 Thunder Epi Microscope	Leica Microsystems	Cat# 53743
Leica DFC9000 GT camera	Leica Microsystems	N/A
Spark Multimode Microplate Reader	Tecan	N/A
Leica DM4B (dark-field microscopy)	Leica Microsystems	Cat# 11888866

### Experimental model and study participant details

#### Ticks

Unfed and pathogen-free nymphal and female *I. ricinus* ticks were obtained from a laboratory colony maintained by the Insect Services GmbH (Berlin, Germany). Upon arrival, the ticks were acclimated at 22°C and 97-100% relative humidity under a 14:10-hour light-dark photoperiod for a minimum of seven days prior to initiation of the feeding experiments. Adult female ticks were primarily used for feeding experiments for practical reasons and due to their improved survival following bacterial exposure compared with nymphs. In addition, host-seeking *I. ricinus* nymphs and females were collected from vegetation at two recreational sites in Vienna, Austria, Steinhofgründe and Lainzer Tiergarten, in May and June 2023 using the flagging method.

#### Bacterial strains

*Staphylococcus epidermidis* LTG-5 and *B. afzelii* RS 163_11i strains were isolated from questing *I. ricinus* ticks collected in Austria^18^ and Serbia,^62^ respectively. As our previous work yielded only Gram-positive isolates,^18^ the laboratory strain *P. putida* KT2440 was obtained from the bacterial collection of the Division of Microbial Ecology (DoME) and used as a representative Gram-negative bacterium. This species was also chosen for experimental tick infections because it had previously been reported in the gut of *Ixodes* ticks.^9^^,^^11^^,^^19^ All experimental procedures were designed and performed in compliance with institutional biosafety regulations and relevant national and international guidelines.

### Method details

#### Oral administration of bacteria via capillary feeding

Bacterial strains used for experimental tick infections were retrieved from glycerol stocks and prepared according to a previously described protocol.^18^ Briefly, *S. epidermidis* and *P. putida* were inoculated into Luria-Bertani (LB) broth (Thermo Fisher Scientific, MA, USA) and incubated overnight at 37°C with orbital shaking (200 rpm). Bacterial cells from overnight cultures were harvested, washed in sterile PBS, and adjusted to a standardized OD_600_ of 0.1. *Borrelia* was grown in Barbour-Stoenner-Kelly (BSK-H) medium supplemented with 6% rabbit serum (Sigma-Aldrich, MO, USA) and maintained under microaerophilic conditions at 33°C until reaching a density of approximately 1 x 10^7^ spirochetes/ml. Quantification and viability of *Borrelia* spirochetes was conducted using dark-field microscopy with a Neubauer counting chamber (Wertheim, Germany).

Sterile 5 μl glass capillaries (Drummond, PA, USA) containing individual bacterial suspensions were placed under a stereomicroscope (Olympus SZ61, Japan) over the hypostomes of female ticks, which were immobilized on double-sided adhesive tape mounted on a microscope slide.^18^ The ticks were then allowed to feed for 1 h in a humidified chamber at 30°C. Control ticks received only PBS. After feeding, they were placed in sterile 5 ml tubes with holes and kept in a desiccator at 22°C and 97-100% relative humidity for 8 h before dissection. In co-infection experiments, ticks were first fed with either *S. epidermidis* or *P. putida*, followed by *B. afzelii*. To ensure that all ticks received an equal number of bacterial cells, 0.6 μl of bacterial suspension was loaded into capillaries. The ticks were allowed to feed until they consumed the entire suspension (45 – 60 min).

#### Tick dissection, RNA extraction, cDNA synthesis, and RT-qPCR

Prior to dissection, the ticks were surface sterilized with 70% ethanol for 1 min and then rinsed three times with sterile water. The guts were aseptically dissected under a stereomicroscope (Olympus SZ61, Japan) using sterile surgical blades size #11 (Integra Miltex, Japan) and fine-tipped forceps.^63^ RNA was extracted from pooled samples of three guts using the Total RNA Purification kit (Norgen Biotek, Ontario, Canada) according to the manufacturer’s instructions. The gut tissue was homogenized in 320 μl of Buffer RL supplemented with β-mercaptoethanol (Sigma-Aldrich, MO, USA) in innuSPEED Lysis Tubes P (IST Innuscreen, Germany) using the FastPrep®-24 5G bead beating grinder and lysis system set to 4.0 m/s for 20 s (MP Biomedicals, CA, USA). RNA quantification was performed using a Qubit 4 Fluorometer and Qubit RNA Broad Range Assay Kit (Thermo Fisher Scientific, MA, USA). The extracted RNA was subsequently treated with DNase (TURBO DNA-free Kit, Ambion, MA, USA) and reverse-transcribed into cDNA using the High-Capacity cDNA Reverse Transcription Kit (Applied Biosystems, MA, USA). The resulting cDNA was diluted 1:1 in nuclease-free water. Target gene expression levels were quantified by RT-qPCR and normalized against the *I. ricinus* elongation factor 1-alpha (*elf-1a*) housekeeping gene, as described previously.^18^ Primer sequences are listed in Table S1. Each sample was analyzed in duplicate and melt curve analysis was conducted to verify the specificity of the generated amplicons.

#### DNA extraction and quantification of bacterial loads in tick guts

Genomic DNA was extracted from individual tick guts using a QIAamp DNA Mini Kit (Qiagen, Germany) after overnight digestion with proteinase K at 56°C. Before dissection, ticks were washed with 70% ethanol and sterile water. The spirochete load was quantified using a probe-based qPCR assay as previously described.^18^ Each sample was analyzed in two technical replicates. Quantification of *Pseudomonas* spp. in field-collected ticks was performed using SYBR Green qPCR with genus-specific primers.^64^ PCRs were carried out using iQ SYBR Green Supermix (Bio-Rad Laboratories, Germany) following the manufacturer’s instructions. The relative bacterial abundance was normalized to the tick mitochondrial 16S rRNA reference gene and calculated using the 2^-ΔΔCt^ method.^65^

#### RNA interference

dsRNA synthesis and tick injection were performed according to the protocol described by Duscher et al.^66^ Briefly, oligonucleotide primers containing T7 promoter sequences at their 5′ ends (TAATACGACTCACTATAGG) were used to amplify a 206 bp long fragment of the *defensin 1* gene from cDNA (Table S1). The resulting PCR product was purified using the Monarch Spin PCR & DNA Cleanup Kit (New England Biolabs, MA, USA) and subsequently used as a template for dsRNA synthesis using the MEGAscript RNAi Kit (Ambion, MA, USA). The reaction was incubated at 37°C for 4 h. The synthesized dsRNA was diluted to a working concentration of 1 x 10^12^ molecules/μl in elution buffer (10 mM Tris-HCl, 1 mM EDTA, pH 7). Fifteen unfed adult female ticks were surface-sterilized and microinjected with approximately 0.5 μl of diluted dsRNA into the hemocoel using a 33-gauge syringe (Hamilton, NV, USA). Control ticks received an equal volume of elution buffer. Following injection, ticks were maintained in a glass desiccator at room temperature for 24 h before being infected with *P. putida* via capillary feeding, as described above. The efficiency of dsRNA-mediated silencing was evaluated 8 h post-feeding by RT-qPCR in pools of three dissected guts.

#### Simultaneous detection of ROS and RNS in tick tissues

ROS and RNS production in tick tissues was measured as previously described.^42^^,^^67^ Briefly, salivary glands and guts were dissected from unfed nymphs or nymphs fed either PBS or bacterial cell suspensions. Tissues were stained on microscope slides with a mixture of 10 μM DHE and 10 μM DAF-2DA fluorescent probes (MedChemExpress, NJ, USA) and incubated for 20 min at room temperature in the dark. Following incubation, tissues were washed three times with sterile PBS and imaged using a Leica DMi8 Thunder Epifluorescence Microscope (Leica Microsystems, Germany). Images were acquired using Leica LAS X software (Leica Thunder Imager, Leica Microsystems, Germany) by scanning brightfield, Cy3 (DHE), and FITC (DAF-2DA) fluorescence channels. Identical exposure settings were applied across samples to enable fluorescence intensity comparisons. The mean fluorescence intensity was measured using ImageJ software.^68^

#### Antibacterial assay

mDef1 was synthesized and purified by GenScript Biotech, Netherlands. Its structure was predicted using the web-based AlphaFold Server platform (alphafoldserver.com). The peptide, with a purity of 90.1%, was dissolved in sterile water to a concentration of 3 mM and stored at - 80°C until use. Antimicrobial activity was evaluated according to a previously described protocol with minor modifications.^29^ Briefly, *S. epidermidis* and *P. putida* strains were streaked onto Mueller-Hinton (MH) agar plates and incubated overnight at 37°C. A single colony from each strain was transferred to MH broth and cultured under the same conditions. The following day, the bacterial suspensions were adjusted in MH broth to an OD_600_ of 0.01 and subsequently diluted 1:1 with fresh medium to obtain a final OD_600_ of 0.005. The peptide stock solution was serially diluted two-fold in MH broth, and 100 μl of each dilution was mixed with 100 μl of the bacterial culture, resulting in final defensin concentrations ranging from 150 μM to 0.0025 μM. Bacterial growth was monitored at 30°C by measuring OD_600_ every 20 min for 24 h under orbital shaking using a Spark Multimode Microplate Reader (Tecan, Switzerland). The sterility of the peptide was verified by testing residual solution remaining in the pipette tip. Bacterial cultures without peptide served as positive controls. The mean OD values of the negative control (MH medium only) at each time point were subtracted from the experimental readings to correct for background absorbance. All experiments were performed in triplicates.

To evaluate the borreliacidal activity of mDef1, we performed *in vitro* antimicrobial susceptibility assay as previously described.^69^ The *B. afzelii* suspension containing approximately 4 x 10^5^ spirochetes/ml was incubated at 33°C for 24 h with different peptide dilutions. After 24 h of incubation, the number of spirochetes, viability, and morphology were evaluated using dark-field microscopy. Quantification of *Borrelia* was also carried out using qPCR, since the formation of bacterial aggregates in mDef-treated cultures hindered reliable cell counting by dark-field microscopy. The spirochete numbers were determined by extrapolation from a standard curve generated from ten-fold serial dilutions of DNA obtained from a pure *B. afzelii* culture, ranging from 10^7^ to 10^0^ spirochetes/ml. DNA was extracted from 100 μl of culture using a QIAamp DNA Mini Kit (Qiagen, Germany). Furthermore, spirochete viability was evaluated using the LIVE/DEAD™ BacLight^TM^ Bacterial Viability Kit (Invitrogen, CA, USA), according to the manufacturer’s instructions, and visualized with a Leica DMi8 Thunder Epifluorescence Microscope (Leica Microsystems, Germany). To evaluate the specificity of mDef1, an additional assay was conducted using its inactivated form. Disulfide bond reduction with DTT was carried out according to the protocol described by Guizzo et al.,^29^ and the *Borrelia* growth inhibition assay was performed as described above. The final concentration of both untreated and DTT-treated mDef1 was 150 μM.

#### Supernatant and bacterial cell lysate preparation

*Pseudomonas putida* was cultured in LB medium at 37°C with orbital shaking at 200 rpm overnight. The culture was then centrifuged at 8,000 rpm for 10 min at 4°C to separate the cells from the medium. The cell-free supernatant was collected and filtered using a 0.22 μm syringe filter. The cell pellet was resuspended in sterile PBS and subjected to three freeze-thaw cycles in liquid nitrogen and a 37°C water bath, followed by three sonication cycles at 35 Hz for 45 s each. The lysate was subsequently centrifuged at 13,000 rpm for 25 min at 4°C to remove cell debris. The resulting supernatant, containing soluble cellular components, was collected and filtered through a 0.22 μm syringe filter for downstream applications.^70^

#### Microbe-microbe interaction analyses

To investigate the potential direct interactions between *P. putida* and *B. afzelii*, 50 μl of the undiluted *P. putida* supernatant or cell lysate was mixed with 150 μl of the *B. afzelii* culture (∼5 x 10^5^ spirochetes/ml). A culture containing 50 μl of PBS served as positive control. The cultures were then incubated for 24 h at 33°C under microaerophilic conditions, after which *Borrelia* load and cell viability were assessed using qPCR and microscopy as previously described. For the *in vitro* coculture experiments, ten-fold serial dilutions (1:10 to 1:10,000) of overnight *P. putida* culture, adjusted to an OD_600_ of 0.1, were prepared in sterile PBS and subsequently mixed with 150 μL of *B. afzelii* culture. Pure cultures of both bacterial species served as positive controls. Following incubation at 33°C for 3 h and 6 h, *Borrelia* load and viability were evaluated using qPCR and microscopy.

### Quantification and statistical analysis

Statistical analyses and graphical representations were performed using GraphPad Prism 10 (GraphPad Software Inc., CA, USA). Data normality was assessed using the Shapiro-Wilk test. For comparisons involving three or more groups with normally distributed data, one-way analysis of variance (ANOVA) with Dunnett’s *post-hoc* test was applied. When normality assumptions were not met, the Kruskal-Wallis test followed by Dunn’s multiple comparisons was used. Comparisons between two groups were conducted using either a two-tailed *t*-test or a non-parametric Mann-Whitney *U* test, depending on the data distribution. Only statistically significant differences are shown in the figures. Differences were considered statistically significant at *p* < 0.05. Data are presented as the mean ± SD based on two or three technical replicates, with the number of replicates specified in the figures.
