# Supplementary material for: Immune-mediated microbial interference governs Borrelia colonization of the tick gut
Source: iScience. 2026 Apr 24;29(5):115628. doi: 10.1016/j.isci.2026.115628 (PMC13127326; doi:10.1016/j.isci.2026.115628)
Supplement: Document S1. Figures S1–S3 and Table S1 [file mmc1.pdf]

iScience, Volume *29*

Supplemental information

Immune-mediated microbial interference governs *Borrelia* colonization of the tick gut

Adnan Hodžić, Martin Kunert, Mia Juračić, Gorana Veinović, Ratko Sukara, Snežana Tomanović, David Seki, and David Berry

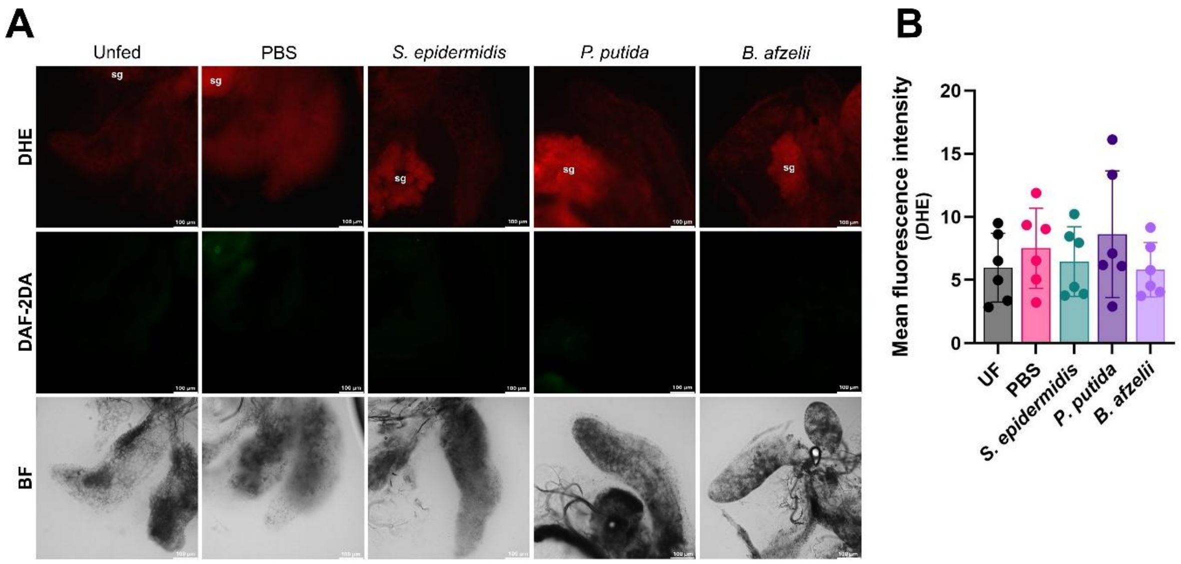

Figure S1. ROS and RNS production in tissues of nymphal ticks.

(A) ROS and RNS levels in dissected salivary glands and midguts from unfed, uninfected (PBS), and infected nymphs were visualized using DHE and DAF-2D fluorescent probes, respectively. sg: salivary glands. Scale-bar: 100 µm.

(B) Mean fluorescence intensity of DHE-stained tick guts was measured using ImageJ software. Each dot represents an individual gut. Statistical significance was assessed using one-way ANOVA followed by Dunnett's post-hoc test.

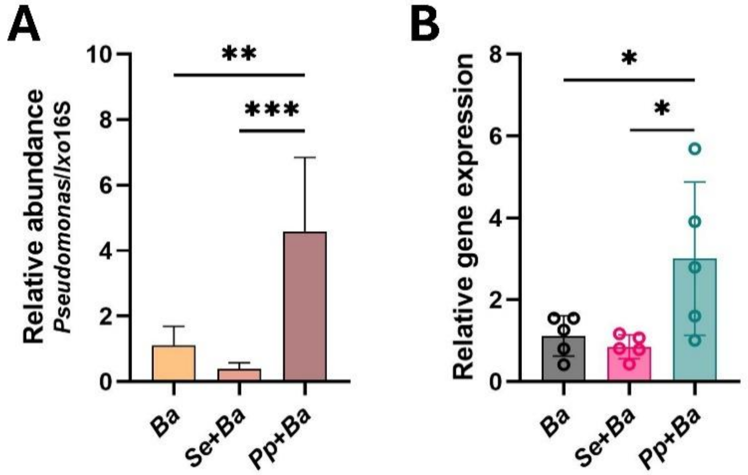

Figure S2. Relative abundance of *Pseudomonas* and *defensin 1* expression levels in co-infected ticks.

(A) Relative abundance of *Pseudomonas* in the guts of female ticks co-infected with *S. epidermidis* (*Se*+*Ba*) or *P. putida* (*Pp*+*Ba*) at 7 h post-*Borrelia* infection, compared with ticks infected with *B. afzelii* alone (*Ba*). Data are shown as mean ± SD of two technical replicates. Statistical significance was determined by one-way ANOVA ($^{**}p < 0.01$; $^{***}p < 0.001$).

(B) Relative expression levels of *defensin 1*, normalized to the tick *elf-1a* gene. Each point represents a pool of three guts. Data are presented as mean ± SD of two technical replicates. Statistical comparisons were performed using one-way ANOVA ($^{*}p < 0.05$).

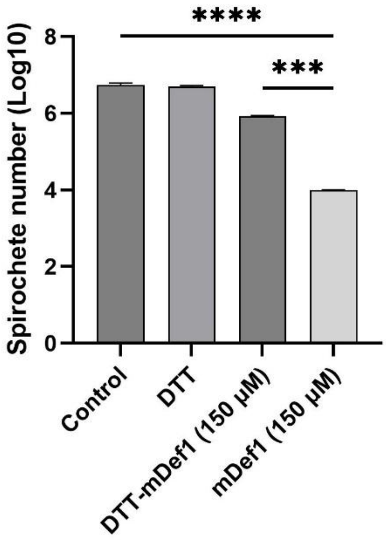

Figure S3. Reduction of disulfide bonds diminishes the inhibitory effect of mDef1 on spirochetes.

Spirochete loads were quantified by qPCR after 24 h of incubation with untreated control, DTT-only control, DTT-treated mDef1, or untreated mDef1. Both untreated and DTT-treated mDef1 were used at the final concentration of 150 µM. Data are shown as mean ± SD of two technical replicates. Statistical significance was assessed by one-way ANOVA ($^{***}p < 0.001$; $^{****}p < 0.0001$).

Table S1. Primers and probes used in the present study.

Target	Name	Sequence (5´ - 3´)	Tm	bp	Reference
**Probe-based qPCR**					
Ixodes 16S	Ixo16S-F<br>Ixo16S-R<br>Ixo16S-Probe	AAAAAAATACTCTAGGGATAACAGCGTAA<br>ACCAAAAAAGAATCCTAATCCAACA<br>HEX- TTTTGGATAGTTCATATAGATAAAATAGTTTGCGACCTCG -BHQ1	60	98	[1]
Borrelia flagellin	FlaF1A<br>FlaR1<br>Fla Probe1	AGCAAATTTAGGTGCTTTCCAA<br>GCAATCATTGCCATTGCAGA<br>FAM- TGCTACAACCTCATCTGTCATTGTAGCATCTTTTATTTG -BHQ1	60	174	[2]
**qPCR**					
Staphylococcus tuf	TstaG422<br>Tstag765	GGCCGTGTTGAACGTGGTCAAATCA<br>TATHACCATTTCAGTACCTTCTGGTAA	60	370	[3]
Pseudomonas 16S	Pseu-F<br>Pseu-R	ACCGCATACGTCCTACGG<br>CGAAGACCTTCTTCACACACG	60	250	[4]
**RT-qPCR**					
elf-1a<br>(E3SS18)	Irelf1a_f<br>Irelf1a_r	CCAAGACCTTTGTTGCTGGC<br>CAGGCTGGTGTCTGCGATAT	63	91	[5]
stat<br>(V5H6X7)	Irstat-F<br>Irstat-R	GAGTATCACCAGGCCACGAG<br>CCACCGCCAACCTTGAATTG	63	149	[5]
myD88<br>(V5GVQ3)	myD88-F<br>myD88-R	TCTGAAGTCCCACGAATGCC<br>CATCCTGATCATGCCGTCGA	63	124	[5]
dorsal<br>(GIXL01005860.1)	dorsal-F<br>dorsal-R	GACGTGCACCTCCTCTTGA<br>CTCCGGATCCCTCTCGTT	63	90	[6]
xiap<br>(V5H7Q7)	xiap-F<br>xiap-R	TGTCACAAGAACGATCGCCA<br>AAACTTGGAGAGTCGCGGAG	63	85	[5]
relish<br>(GFVZ01045168.1)	rel2-F<br>rel2-R	ACCCTCTGCTGCGTCTACTC<br>TCCTCGTCCTCCTCAAAGAA	63	173	[7]
duox<br>(A0A147BNH4)	duox-F<br>duox-R	AGCTGTACCACGACAACCTG<br>TTCTCGAACCAGAAGCGGTC	63	148	This study
nos<br>(A0A147BLV2)	nos-F<br>nos-R	GAAGTGGAGCGTCAGGTTCA<br>AACACCTGCAGTTTGGACCA	63	134	[5]
nox<br>(GIDG01041772.1*)	nox-F1<br>nox-R1	TGATACAGCCGAACGAGCTC<br>AGCTGATGGAGTTGGTGTCG	63	141	This study
sod<br>(A0A0K8RHC8)	sod-F1<br>sod-R1	GTGATTGCTGGCAACGATGG<br>CGTCCAATAATGCTGTGCGG	63	86	This study
cat<br>(A0A131XP47)	cat-F<br>cat-R	GACCGCAACCCCAAGAACTA<br>GAACAGACGACCCTGAAGCA	63	114	This study

defensin 1<br>(Q7YXK5)	def1-F<br>def1-R	GGTGGCTACTACTGCCCATTTTTT<br>TCAGACGCAGATGCAGGTCTTTT	63	114	[8]
defensin 4<br>(A0A089VKM9)	Irdef4-F<br>Irdef4-R	CGCCGACTTTTCAAACGACA<br>ATGGGCAGTAGTAACCACCG	63	95	[5]
micropulsin<br>(A0A0K8RB39)	micropls-F<br>micropls-R	AAGTCGAAAAGCAGGTGGGT<br>GCCGCCGTAAAATGCTTCTT	63	103	[9]
peritrophin 1<br>(V5H8Q8)	per1_F<br>per1_R	CACCGAGCCGTCTTGTCC<br>ACTTTCCGGTGACCTTGTTGA	63	152	[5]
mucin<br>(A0A0K8R6Q9)	per2_F<br>per2_R	GCTGTGGCCAAGGACTTCA<br>CCACCTTGACGCTGATCTTGT	63	151	This study
**RNAi**					
defensin 1<br>(Q7YXK5)	T7def1-F<br>T7def1-R	TAATACGACTCACTATAGGCATGAAGGTCCTTGCCGTCT<br>TAATACGACTCACTATAGGTTTTGAGAAAACCGCCGCAG	65	206	This study

*Primers were designed based on the *nox* sequence derived from an *I. ricinus* transcriptome (NCBI Project Nos.: PRJNA595586)

Supplemental references

S1. Becker, N.S., Rollins, R.E., Stephens, R., Sato, K., Brachmann, A., Nakao, M., and Kawabata, H. (2023). *Candidatus* Lariskella arthopodarum endosymbiont is the main factor differentiating the microbiome communities of female and male *Borrelia*-positive *Ixodes persulcatus* ticks. Ticks Tick Borne Dis. 14, 102183. https://doi.org/10.1016/j.ttbdis.2023.102183.

S2. Schwaiger, M., Péter, O., and Cassinotti, P. (2001). Routine diagnosis of *Borrelia burgdorferi* (sensu lato) infections using a real-time PCR assay. Clin. Microbiol. Infect. 7, 461–469. https://doi.org/10.1046/j.1198-743x.2001.00282.x.

S3. Wampach, L., Heintz-Buschart, A., Hogan, A., Muller, E.E.L., Narayanasamy, S., Laczny, C.C., Hugerth, L.W., Bindl, L., Bottu, J., Andersson, A.F., de Beaufort, C., and Wilmes, P. (2017). Colonization and succession within the human gut microbiome by archaea, bacteria, and microeukaryotes during the first year of life. Front. Microbiol. 8, 738. https://doi.org/10.3389/fmicb.2017.00738.

S4. Najafpour, B., Pinto, P.I.S., Canario, A.V.M., and Power, D.M. (2022). Quantifying dominant bacterial genera detected in metagenomic data from fish eggs and larvae using genus-specific primers. MicrobiologyOpen 11, e1274. https://doi.org/10.1002/mbo3.1274.

S5. Hodžić, A., Veinović, G., Alić, A., Seki, D., Kunert, M., Nikolov, G., Sukara, R., Šupić, J., Tomanović, S., and Berry, D. (2024). A metalloprotease secreted by an environmentally acquired gut bacterium hinders *Borrelia afzelii* colonization in *Ixodes ricinus*. Front. Cell. Infect. Microbiol. 14, 1476266. https://doi.org/10.3389/fcimb.2024.1476266.

S6. Jalovecka, M., Malandrin, L., Urbanova, V., Mahmood, S., Snebergerova, P., Peklanska, M., Pavlasova, V., Sima, R., Kopacek, P., Perner, J., and Hajdusek, O. (2024). Activation of the tick Toll pathway to control infection of *Ixodes ricinus* by the apicomplexan parasite *Babesia microti*. PLoS Pathog. 20, e1012743. https://doi.org/10.1371/journal.ppat.1012743.

S7. Narasimhan, S., Schuijt, T.J., Abraham, N.M., Rajeevan, N., Coumou, J., Graham, M., Robson, A., Wu, M.J., Daffre, S., Hovius, J.W., and Fikrig, E. (2017). Modulation of the tick gut milieu by a secreted tick protein favors *Borrelia burgdorferi* colonization. Nat. Commun. 8, 184. https://doi.org/10.1038/s41467-017-00208-0.

S8. Chrudimská, T., Slaninová, J., Rudenko, N., Růžek, D., and Grubhoffer, L. (2011). Functional characterization of two defensin isoforms of the hard tick *Ixodes ricinus*. Parasit. Vectors 4, 63. https://doi.org/10.1186/1756-3305-4-63.

S9. Urbanová, V., Lu, S., Kalinová, E., Martins, L., Kozelková, T., Dyčka, F., Ribeiro, J.M., Hajdušek, O., Perner, J., and Kopáček, P. (2024). From the fat body to the hemolymph: Profiling tick immune and storage proteins through transcriptomics and proteomics. Insect Biochem. Mol. Biol. 165, 104072. https://doi.org/10.1016/j.ibmb.2024.104072.
